# Unraveling the choice of the north Atlantic subpolar gyre index

**DOI:** 10.1038/s41598-020-57790-5

**Published:** 2020-01-22

**Authors:** Vimal Koul, Jan-Erik Tesdal, Manfred Bersch, Hjálmar Hátún, Sebastian Brune, Leonard Borchert, Helmuth Haak, Corinna Schrum, Johanna Baehr

**Affiliations:** 1 Institute of Oceanography, Center for Earth System Sustainability, Universität Hamburg, Hamburg, Germany; 2 International Max Planck Research School on Earth System Modelling, Max Planck Institute for Meteorology, Hamburg, Germany; 3 Helmholtz-Zentrum Geesthacht, Centre for Materials and Coastal Research, Institute for Coastal Research, Geesthacht, Germany; 4 Lamont-Doherty Earth Observatory, Columbia University, Palisades, New York USA; 5 Faroe Marine Research Institute, Tórshavn, Faroe Islands; 6 Max Planck Institute for Meteorology, Hamburg, Germany; 7 Sorbonne Universités (SU/CNRS/IRD/MNHN), LOCEAN Laboratory, Institut Pierre Simon Laplace (IPSL), Paris, France

**Keywords:** Physical oceanography, Atmospheric dynamics

## Abstract

The north Atlantic subpolar gyre (SPG) has been widely implicated as the source of large-scale changes in the subpolar marine environment. However, inconsistencies between indices of SPG-strength have raised questions about the active role SPG-strength and size play in determining water properties in the eastern subpolar North Atlantic (ENA). Here, by analyzing various SPG indices derived from observations and a global coupled model, we show that the choice of the SPG index dictates the interpretation of SPG strength-salinity relationship in the ENA. Variability in geostrophic currents derived from observed hydrography and model based Lagrangian trajectories reveal zonal shifts of advective pathways in the ENA and meridional shifts in the western intergyre region. Such shifts in advective pathways are manifestations of variability in the size and strength of the SPG, and they impact salinity by modulating the proportion of subpolar and subtropical waters reaching the ENA. SPG indices based on subsurface density and principal component analysis of sea surface height variability capture these shifts in advective pathways, and are therefore best suited to describe SPG-salinity relationship in the ENA. Our results establish the dynamical constraints on the choice of the SPG index and emphasize that SPG indices should be cautiously interpreted.

## Introduction

The northward transport of heat and salt in the North Atlantic is an important aspect of climate variability as it determines the exchanges of heat and salt with the Arctic Mediterranean. Of particular importance is the interannual to decadal variability in salinity of waters headed towards the sites of deep convection. This is because salinity affects buoyancy and density stratification, and can thus influence the formation of dense waters which form the lower limb of the thermohaline circulation. In the eastern subpolar North Atlantic (ENA, 50°N to 60°N, 15°W to 30°W), the source of interannual to decadal variability in salinity has been a subject of various investigations, and the variability in the North Atlantic Subpolar Gyre (SPG) circulation has emerged as the leading cause. Observations of temperature and salinity suggest that the North Atlantic Oscillation (NAO) drives changes in the size and strength of the SPG^1,2^, and that these changes are manifested in the properties of the ENA^3,4^. Although variability in the size and strength of the SPG may not always be attributable to the NAO^5,6^, it has repeatedly been linked to salinity changes in the ENA^7,8^, leaving a minor role for local air-sea fluxes^9^ and the Atlantic Meridional Overturning Circulation (AMOC)^10,11^.

In contrast to this abundant evidence, recent investigations^12,13^ do not find any relationship between SPG size/strength and salinity in the ENA. At the heart of this discrepancy are (a) the different ways of defining SPG strength and (b) the role of the NAO in driving changes in SPG circulation. The widely-used sea surface height (SSH) based Gyre Index^5,7^, which is linked to salinity changes in the ENA, does not account for the sea level rise during the altimeter period^13^, and is therefore not an appropriate proxy for the size or strength of the SPG. A new index^13^ of SPG size and strength based on the largest closed contours of SSH, which accounts for the trend in the SSH data, does not project onto the NAO and does not show any significant relationship with salinity in the ENA at interannual timescales. Moreover, large variations in salinity in the ENA, for example, those seen in the 1990s, are thought to be primarily linked to abrupt changes in local oceanic convergence, due to the wind-driven fast response of oceanic circulation to abrupt changes in the NAO state^12,14,15^. This is in contrast to connecting salinity variations in the ENA to buoyancy-driven strengthening of SPG circulation.

The conflicting views on SPG variability also involve the question of causality. On the one hand the atmosphere-driven variability in the size and strength of the SPG, proxied by the Gyre Index, has been shown to precede hydrographic variability in the ENA^7,10,16^, while on the other hand the promising results on the SPG predictability (which includes the ENA)^17,18^ point towards the dominant role of meridional heat and salt transport in influencing both the SPG strength and salinity in the ENA. Therefore, a mechanistic understanding of the SPG-associated variations in the ENA is necessary in order to ascertain whether different SPG indices, which determine causal relationship of SPG-strength with hydrographic variability in the ENA, emphasize regional or basin scale circulation variability.

These disagreements raise the following questions: Is there a robust relationship between SPG circulation and salinity in the ENA? Put differently, does the pathway and proportion of subtropical and subpolar water masses entering the ENA depend on the size and strength of SPG circulation? It is also unclear which index of the SPG strength should be used for examining the relationship of SPG circulation with physical and biogeochemical variability in the ENA and downstream of the Greenland-Scotland ridge. Thus, another aim of our study is to determine which index (or indices) best represents SPG-salinity relationship. Ambiguity in the definition of SPG index has resulted in multiple SPG indices being used for investigations while keeping the question of underlying mechanisms behind such indices unanswered^19^.

We address the ambiguities highlighted above by examining both the barotropic and baroclinic nature of SPG circulation. This is done by examining SSH based (PC1 SSH, PC2 SSH and FKL), density-based (DENS) and barotrophic streamfunction-based (BTSF) indices of SPG strength. Detailed definitions of all SPG indices are provided in the Methods section. Since a suitably long time series of SSH data before 1993 is not available, and in order to examine the relationship between SPG strength and salinity in a dynamically consistent setting, we complement our analysis of observational data with results from the Max Planck Institute Earth System Model, run in its low resolution setup (MPI-ESM-LR, see Methods). Idealized Lagrangian experiments with MPI-ESM-LR allow us to clarify the SPG strength-salinity relationship and illuminate inconsistencies among SPG indices.

## Results

### Observed SPG strength-salinity relationship

**Figure 1:**
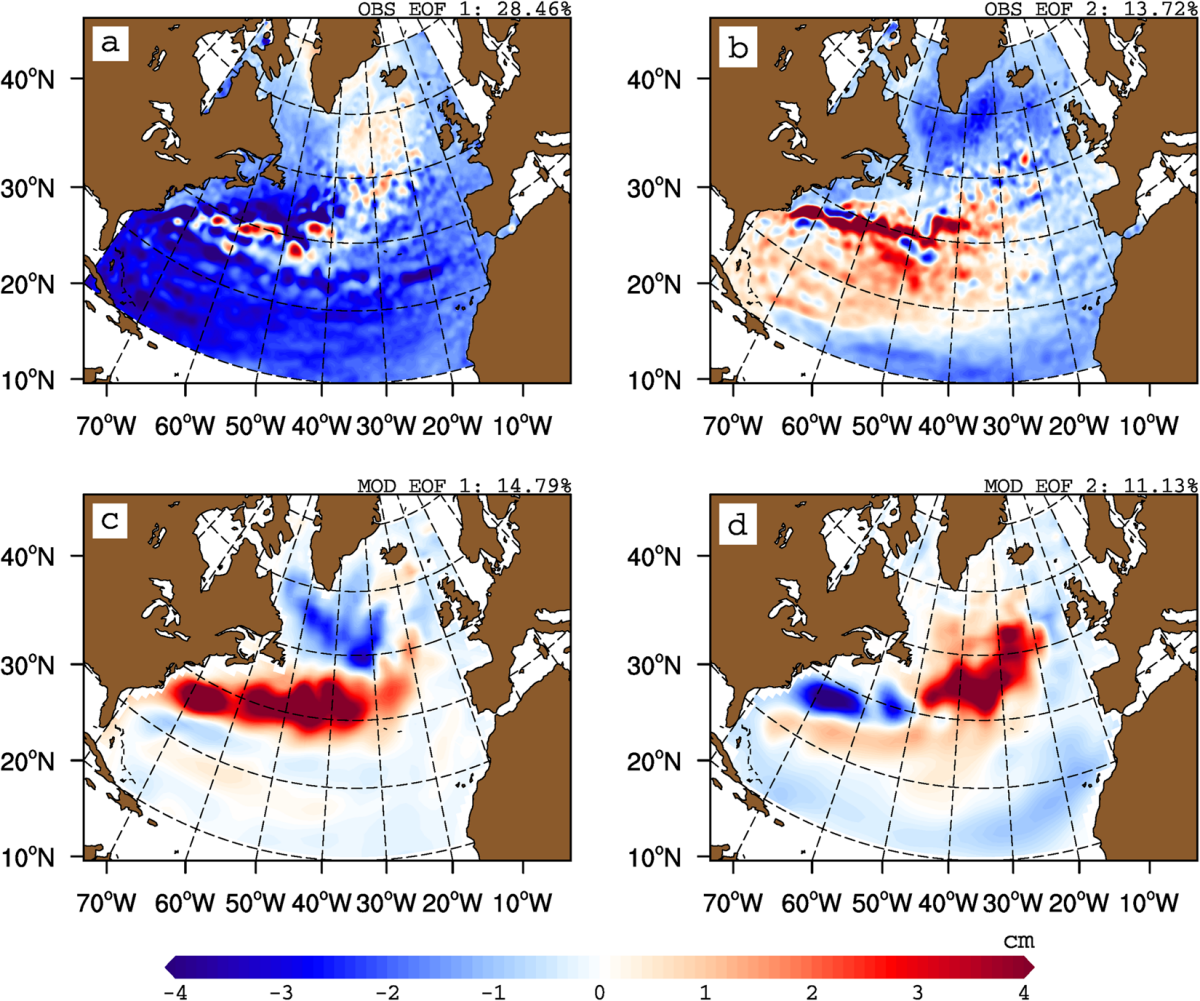
First and second empirical orthogonal function of sea surface height from non-detrended (**a**,**b**) observations (1993–2016) and (**c**,**d**) coupled model (last 200 years) respectively.

**Figure 2:**
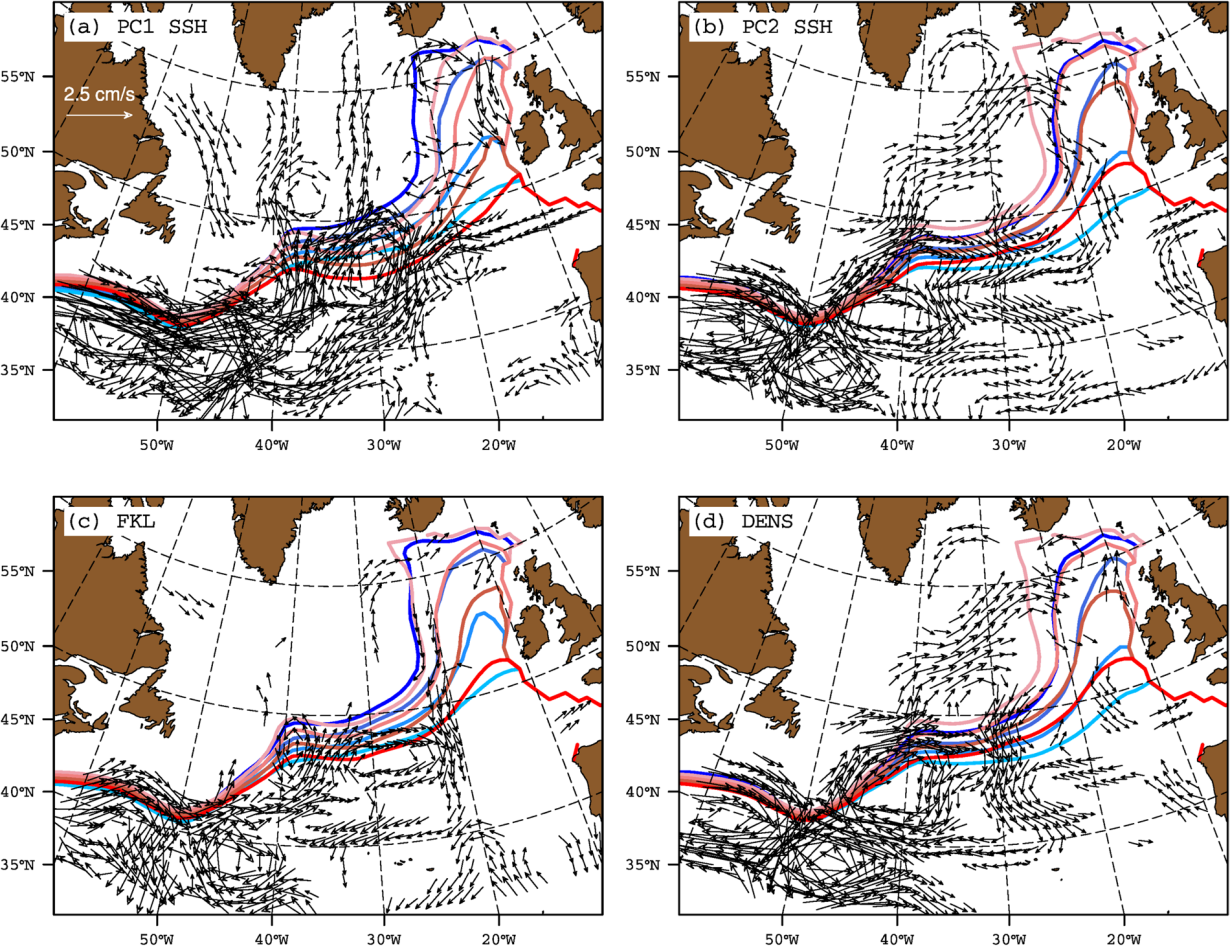
Strong (blue) and weak (red) composites of annual mean top 500 m depth averaged EN4 salinity contours (35.20, 35.30, 35.40 and 35.50 psu) based on strong and weak phases of observed (**a**) PC1 SSH, (**b**) PC2 SSH, (**c**) FKL and (**d**) DENS. The SPG indices are not detrended prior to computing composites. Years for compositing are chosen from 1993–2016 and are based on  ± 0.5 standard deviation of the respective index. Current vectors denote composites difference (strong-weak) of annual mean upper 500 depth averaged geostrophic currents (cm/s). For clarity, only those geostrophic current vectors with magnitude ≥0.5 cm/s are shown.

**Figure 3:**
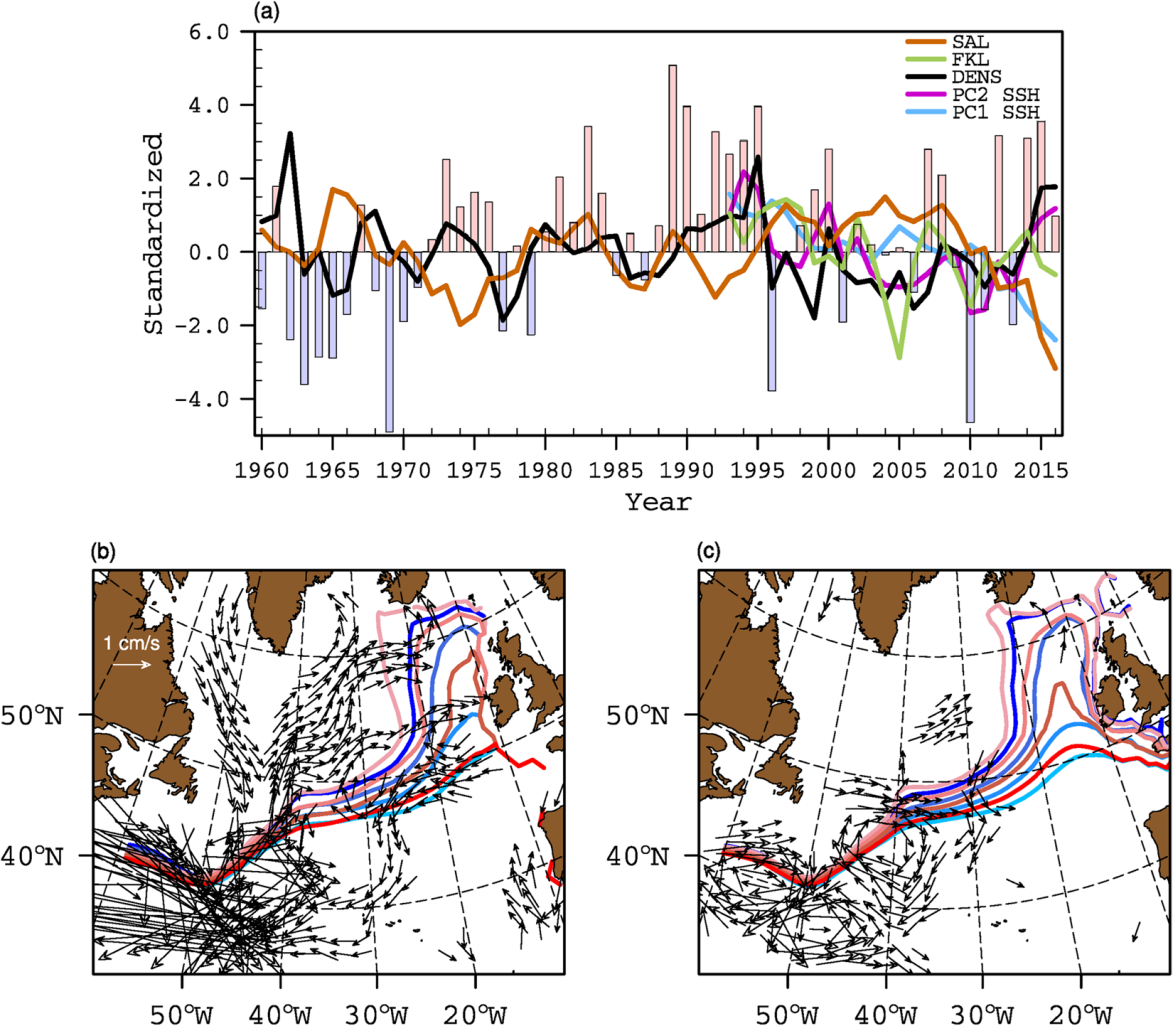
(**a**) Time series of observed SPG indices, salinity (SAL) in the upper 500 m in the ENA (lines) and the NAO (bars). For all SPG indices, positive values represent a strong SPG. (**b**) Strong (blue) and weak (red) composites of annual mean top 500m depth averaged salinity contours (35.20, 35.30, 35.40 and 35.50 psu) based on strong and weak phases of NAO: 1964–68 for NAO −  and 1991–1995 for NAO+. Current vectors denote composites difference (strong-weak) of annual mean upper 500 depth averaged geostrophic currents (cm/s). For clarity, only those geostrophic current vectors with magnitude ≥0.5 cm/s are shown. (**c**) same as (**b**) but for composites based on DENS.

For the altimeter period (1993–2016), SSH data is often considered as a proxy for the observed variability in the SPG circulation, and can therefore be used to assess the relationship between SPG strength and salinity in the ENA. The patterns of the first two modes of SSH variability in the North Atlantic emphasize different spatial characteristics (Fig. 1a,b). While the first mode displays negative weights over most of the North Atlantic, the second mode resembles the well known dipole mode, usually associated with the NAO forcing. A pronounced zonal band of strong positive weights is also present in the western subtropical gyre at 40°N latitude. The relationship between the first principal component of SSH variability in the North Atlantic (PC1 SSH) and salinity in the ENA suggests that with decreasing SSH, salinity in the ENA increases (Fig. 2a). However, the presence of a trend in PC1 SSH (Fig. 3a) together with weak spatial weights in the Irminger Sea and Iceland Basin (Fig. 1a) suggests that PC1 SSH does not represent variability in SPG strength. The second mode of SSH variability shows strong negative weights in the Irminger Sea and Iceland Basin (Fig. 1b), its associated time series (PC2 SSH) does not have a monotonic trend (Fig. 3a), and the relationship between PC2 SSH and salinity suggests that freshening in the ENA is concomitant with a strong SPG (Fig. 2b). Isohalines shift eastward and southward in the ENA when SSH decreases in the Irminger Sea and Iceland Basin.

The index based on the largest closed contours of SSH (hereafter FKL^13^), does not suggest latitudinally coherent shifts in isohalines in the ENA in response to changes in SPG strength (Fig. 2c). This contrasts with the response of salinity to PC2 SSH (Fig. 2b). The FKL index also does not show any shifts in the SPG boundary defined as the largest closed contour of altimeter SSH^13^. However, the density-based SPG index (hereafter DENS^20^), used as a proxy for changes in baroclinic SPG circulation for the period 1993–2016, reveals that the response of upper ocean salinity in the ENA is similar to its response to the PC2 SSH (Fig. 2b,d). Dense upper layers deepen the SPG bowl, which spins up the SPG through geostrophic balance, and causes the isohalines to shift eastwards in the ENA. Therefore, DENS also suggests a latitudinally coherent increase or decrease in salinity with changes in SPG strength.

The strong relationship between the NAO and subpolar North Atlantic circulation has also served as a good proxy for variability in SPG circulation^3,21,22^. During extended periods of positive NAO (NAO+), strong atmospheric forcing across the entire SPG leads to doming of isopycnals (i.e. a decrease in SSH) and thus spinning up the SPG circulation. The opposite happens during extended periods of negative NAO (NAO−). There is an inverse relationship between subsurface density in the central SPG and upper 500 m salinity in ENA during extended periods of persistent NAO forcing (Fig. 3a). This inverse response of upper ocean salinity to cases of extended periods of NAO+ (1991–1995) and NAO −  (1964–1968) is revealed through shifts in isohalines in the ENA (Fig. 3b). Salinity increases coherently at each latitude in the ENA during a NAO −  state and decreases during a NAO+ state. This result is also corroborated by the consistent relationship between DENS and salinity in ENA for the extended time period of 1960–2016 (Fig. 3c).

Changes in the intensity and position of the constituent currents of SPG circulation highlight underlying mechanisms which drive shifts in isohalines in the ENA (Fig. 2). The pattern of baroclinic current anomalies suggests that shifts in isohalines in the ENA are linked to the intensification of the cyclonic circulation in the Irminger Sea and Iceland Basin, which is clearly revealed by PC2 SSH and DENS but not by PC1 SSH and FKL (Fig. 2). This is a key distinction as far as the shifts in isohalines in the ENA are concerned because very similar current anomalies emerge as a result of NAO variability (Fig. 3b). Thus, PC2 SSH and DENS are reflecting the NAO-driven changes in SPG circulation. Higher heat loss during NAO+ phases increases the density and lowers the SSH in the northern regions. The doming of isopycnals due to a strong wind stress curl associated with NAO+ also lowers the SSH. As a consequence, the baroclinic part of the Irminger, Iceland and Rockall Trough branch strengthens during NAO+ phases. The presence of an anticyclonic circulation anomaly in the southwestern intergyre region suggests a northward shift of the North Atlantic Current (NAC) during NAO+ conditions. Thus, the transport of fresh subpolar water from the western SPG is enhanced, which likely causes the eastward shift in isohalines observed during NAO+ conditions. In addition, the anomalous southwestward current south of ENA restricts the northward penetration of subtropical water, further complimenting the freshening in the ENA (Fig. 3b).

In the case of FKL, along with a weak response in the Irminger Sea, Iceland Basin and the Rockall Trough, the northward shift of the NAC is not represented. Thus, the transport of fresh waters from the western SPG, along the northern flank of the NAC and towards the eastern SPG, is not represented by FKL, and consequently the shifts in isohalines are not realized in the ENA using this index. In summary, observed PC2 SSH, DENS and NAO indices suggest that when the SPG is strong, salinity in the ENA decreases and vice versa, and that there are latitudinally coherent shifts in isohalines. All indices, except PC1 SSH and FKL, relate freshening in the ENA to strong cyclonic circulation anomalies in the northeastern subpolar basins, and to anticyclonic anomalies in the southwestern intergyre region.

### Modelled SPG strength-salinity relationship

**Figure 4:**
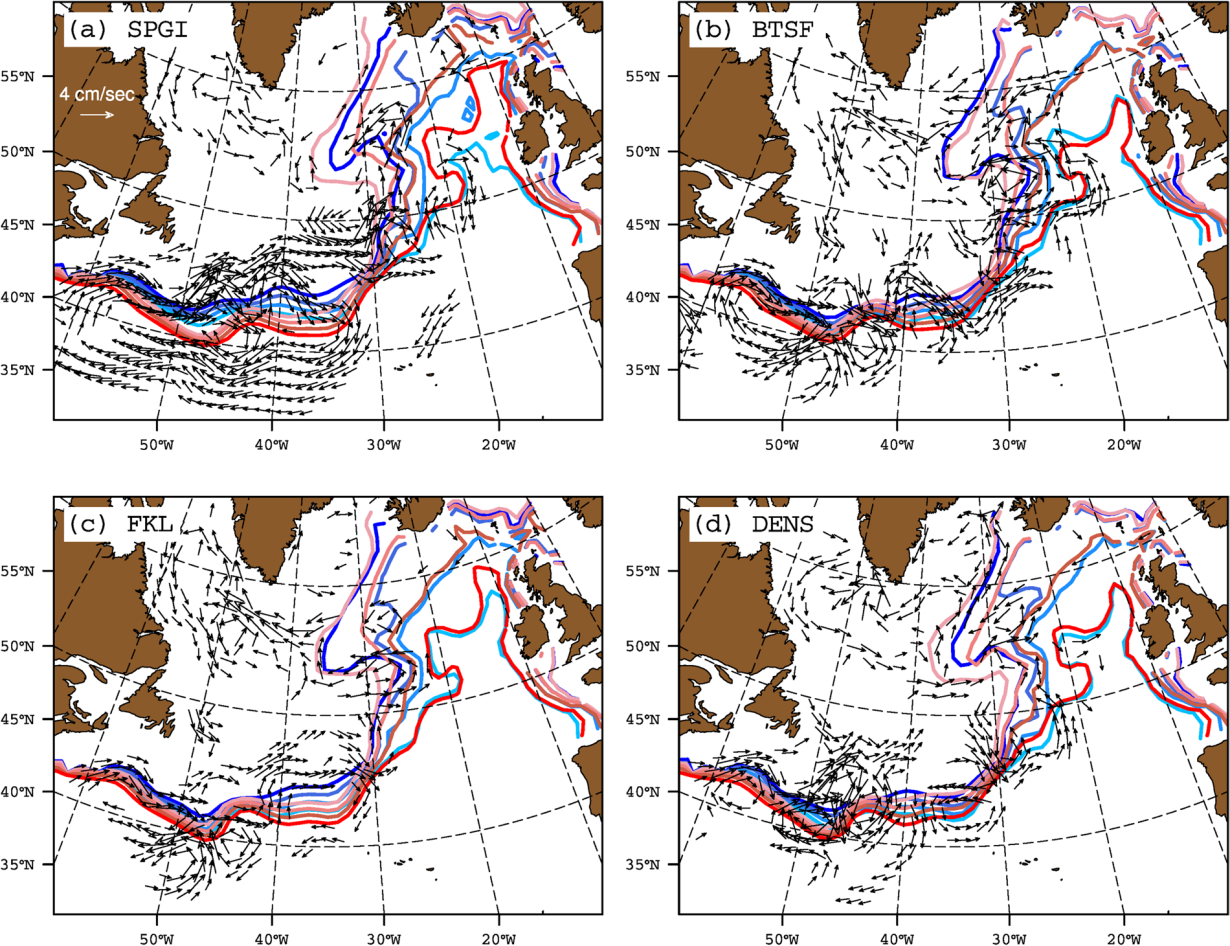
Strong (blue) and weak (red) composites of annual mean top 500 m depth averaged salinity contours (35.20, 35.30, 35.40 and 35.50 psu) based on strong and weak phases of modelled (**a**) SPGI, (**b**) BTSF, (**c**) FKL and (**d**) DENS. The SPG indices are not detrended prior to computing composites. Years for compositing are chosen from last 200 years of the preindustrial control simulation, and are based on  ± 1 standard deviation of the respective index. Current vectors denote composites difference (strong-weak) of annual mean upper 500 depth averaged currents (cm/s). For clarity, only those current vectors with magnitude ≥1.4 cm/s are shown.

The free model simulation does not exhibit a trend during the period of simulation. As a result there is no equivalent of first mode of observed SSH variability in the model. The dipole pattern of the first mode of modelled SSH variability matches the second mode of observed SSH variability, both of which have negative weights in the SPG and positive weights along the NAC path (Fig. 1). Still, there are some differences: the strong positive weights in the western intergyre region are distributed over much larger domain than in the observations. The negative weights in the SPG are also weaker in the north and stronger in the south. We come back to these differences later. The associated principal component (hereafter SPGI) shows latitudinally coherent shifts in isohalines in the ENA (Fig. 4). However, the SPG strength-salinity relationship depicted by SPGI does not match its observational counterpart but has similarities with the observed relationship depicted by PC2 SSH, DENS and NAO. The meridional shifts in isohalines in western intergyre region also emerge clearly in case of SPGI. Therefore, SPGI suggests that a strong SPG has a more zonal orientation than that of a weak SPG. Apart from the SPGI, other indices (see Methods and Table S1), BTSF and FKL do not show any coherent SPG-salinity relationship, while DENS suggests consistent shifts of isohalines in the central SPG region but only subtle shifts in the ENA.

The modelled SPG-salinity relationship depicted by SPGI (i.e., modelled PC1 SSH) matches the observed SPG-salinity relationship depicted by NAO, PC2 SSH and DENS. Therefore, it is of interest to determine if circulation anomalies associated with this modelled index bear any similarity with the observed anomalies. To examine this, we analyzed composites of upper 500 meter currents in the whole North Atlantic computed from weak and strong phases of modeled SPG indices (Fig. 4a). The intensification of cyclonic circulation in the Irminger Sea and Iceland Basin, as emphasized by observed SPG indices, does not determine modelled isohaline shifts in the ENA. However, the anomalous circulation pattern in the southwestern intergyre region emerges as the main circulation anomaly which is related to shifts in isohalines in this region and in the ENA. Furthermore, a salt budget in the ENA clearly suggests that oceanic advection drives salinity variability in the ENA and the role of freshwater flux from the atmosphere is less important in this simulation (Fig. S1).

The modelled indices of SPG strength also exhibit regional differences. For example, the anticyclonic circulation anomaly in the western intergyre region is very clearly revealed by the SPGI and not by the modelled DENS, BTSF and FKL indices (Fig. 4). In the western SPG (west of 40°W), all indices show intensification of the cyclonic circulation during strong index years but BTSF and FKL indices emphasize this intensification more strongly than SPGI and DENS. As discussed later, such differences in modelled SPG indices lead to differences in their relationship with salinity in the ENA.

In summary, the SPG index based on the leading mode of modelled SSH variability (i.e. SPGI) shows latitudinally coherent response of salinity in the ENA to changes in SPG strength; DENS shows changes in salinity as well but only in the central SPG, while BTSF and FKL do not show any latitudinally coherent changes in salinity, although they clearly occur and are ocean-driven, as revealed by the salinity budget (Fig. S1).

### Lagrangian view of modelled SPG circulation variability

**Figure 5:**
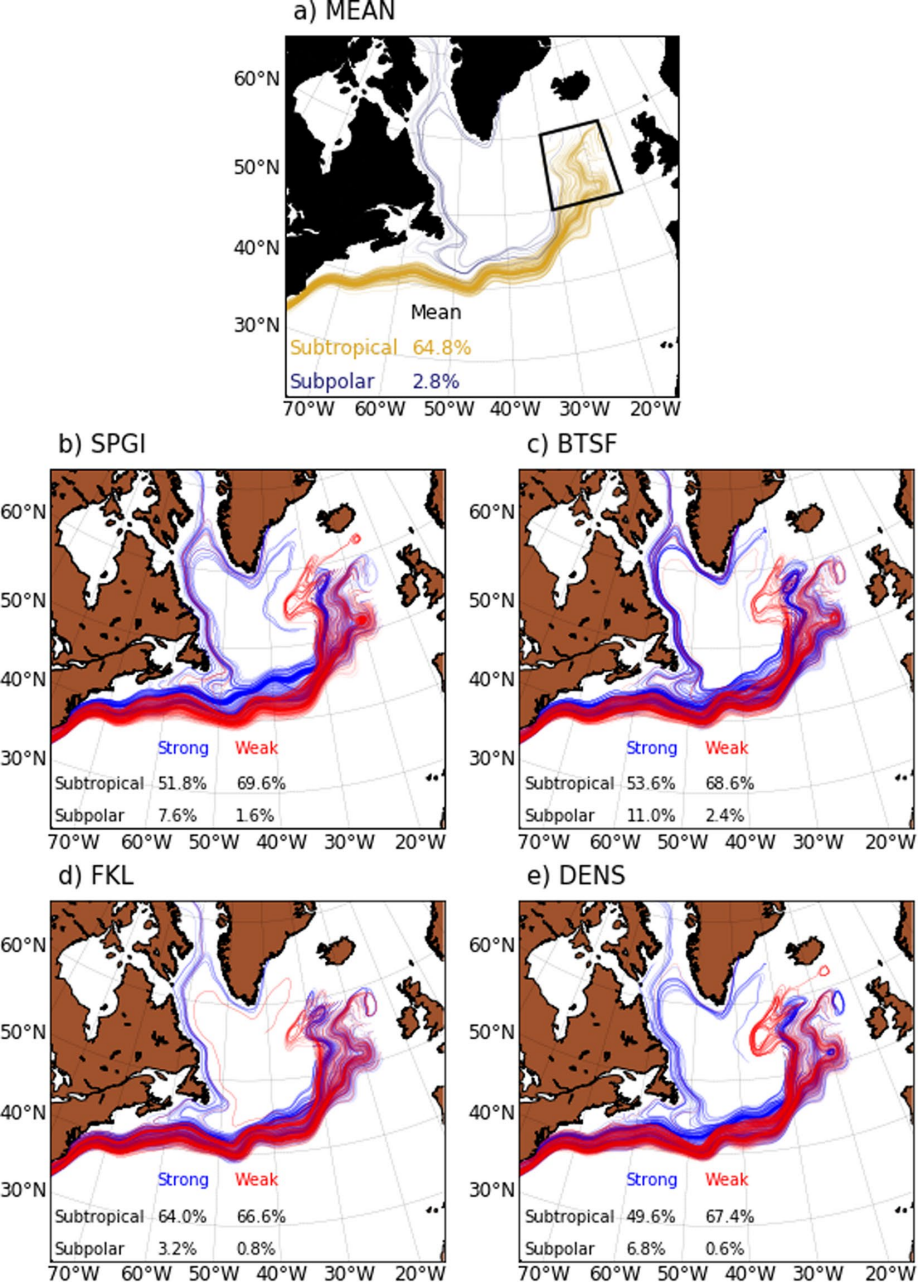
Four year backward trajectories of all virtual floats released in the eastern SPG during (**a**) MEAN circulation conditions and during strong (blue) and weak (red) phases of (**b**) SPGI, (**c**) BTSF, (**d**) FKL and (**e**) DENS. To highlight main features, the trajectory of floats that remain in the ocean throughout their lifetime are drawn by thick lines. The black box in (**a**) shows the region where 500 virtual floats were released in the upper 100 meter. The subtropical floats are defined^23^ as those floats which move south of 32°N at-least once in their lifetime, while the subpolar floats are defined as those floats which move west of 45°W and north of 60°N at-least once in their lifetime of 4 years.

The results from the composite analysis shown above present a rather static picture of the impact of variability in SPG circulation on salinity in the ENA. Also further insight is sought on the significance of latitudinal shifts of the NAC in western intergyre region. One way to reveal water mass pathways during distinct circulation regimes in the North Atlantic is by analyzing Lagrangian trajectories of virtual floats released in the modelled velocity fields (see Methods). Such view of circulation variability is provided by 4-year backward trajectories of virtual floats released in the upper 100 m in the ENA (Fig. 5). First, close to earlier estimates^23^ of the proportion of subtropical and subpolar floats arriving in the ENA, in the present simulation, subtropical floats constitute  > 60% while subpolar floats constitute  < 10% of the total floats (Fig. 5). Second, compared to the mean, all indices unambiguously suggest that the number of subtropical floats arriving in the ENA decreases and the number of subpolar floats increases when the SPG circulation is strong. This implies that a strengthened SPG impedes the subtropical throughput and enhances the subpolar throughput into the ENA.

A noticeable feature that emerges from the comparison of backward trajectories is the shift in advective pathway in the western intergyre region. The largest variability in the latitudinal position of the trajectories passing though the western intergyre region is seen in the case of SPGI followed by DENS. This matches well with the largest response of salinity to changes in SPG circulation shown by SPGI followed by DENS. The northward shift of float trajectories in the intergyre region for strong SPGI also corroborates the result from composite analysis of circulation changes which revealed strong anticyclonic circulation anomalies and relatively large meridional shifts in isohalines in the intergyre region.

In the ENA ( ~ 30°W), the trajectories broaden and shift to the west when the SPG weakens (Fig. 5). Except FKL, such a shift in the ENA is represented by other three modelled indices, suggesting that the shift of trajectories in the ENA is a robust feature. Note that the variation in the proportion of subpolar and subtropical floats does not lead to variation in salinity in the ENA in the case of BTSF and FKL, but does in the case of SPGI and DENS, therefore, it implies that along with the proportion of floats, their pathways also influences salinity. As discussed in the following sections, the meridional shifts in advective pathways in the intergyre region and the zonal shifts in the ENA are manifestations of the expansion and contraction of the SPG.

### Role of large scale atmospheric forcing

**Figure 6:**
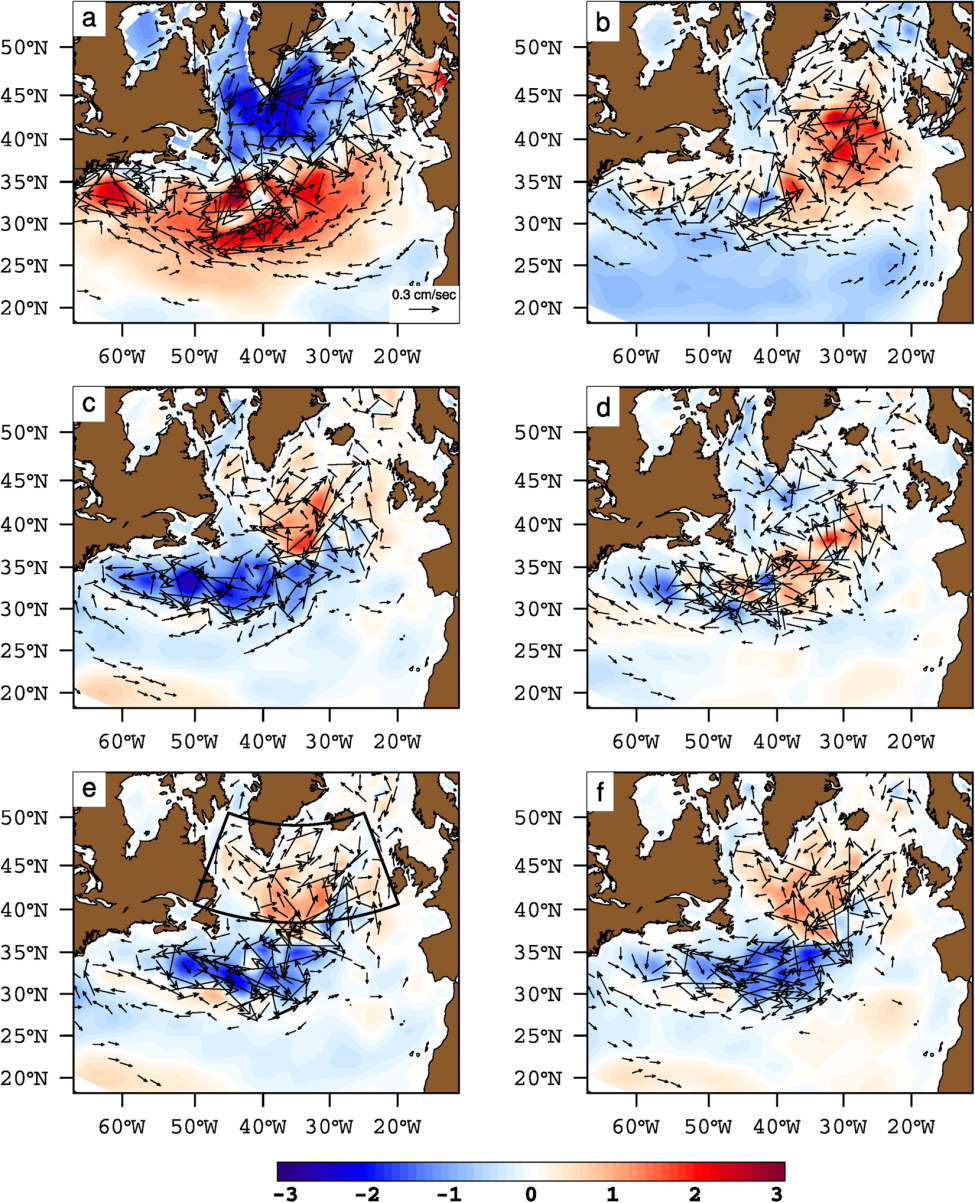
Lag-0 regression of modelled SSH and currents (upper 500 m) on (**a**) NAO, (**b**) EAP index and Lag-10 regression of SSH and currents (upper 500 m) on leading (**c**) NAO, (**d**) EAP, (**e**) average wind stress curl and (**f**) average surface heat flux (latent + sensible) over the SPG (black box). Shown are regression coefficients (units/*σ*(index)). For clarity, only those current vectors with magnitude ≥0.1 cm/sec are shown. NAO—North Atlantic Oscillation, EAP—East Atlantic Pattern.

Up until now we have illustrated that the proportion of subtropical and subpolar floats arriving in the ENA is linked to the state of the SPG circulation as represented by various indices. Now we attend the question of causality. First, the instantaneous relationship between the SSH and large scale atmospheric variability is considered. The instantaneous response of the SSH to the NAO is a decrease in the subpolar latitudes and an increase in the subtropical latitudes (Fig. 6a). This spatial pattern is the characteristic basin scale dipole pattern of the ocean’s response to short term atmospheric variability. However, the instantaneous response of SSH to the East Atlantic Pattern, the second mode of modelled sea level pressure variability, is zonally asymmetric (Fig. 6b). Its influence is larger in the ENA than in the western SPG. Nevertheless, none of these regression patterns match the spatial pattern of the leading mode of modelled SSH variability (Fig. 1c).

When the lagged (decadal) response of the modelled SSH field to the NAO is considered, it emerges that the spatial pattern of the leading mode of modelled SSH variability mirrors the spatial pattern of the lagged response (Fig. 6c). A strong SPG circulation, driven by the NAO, turns into a weak SPG circulation within a decade, suggesting a negative feedback. Such a dipole SSH pattern does not emerge as a lagged response to the East Atlantic Pattern (Fig. 6d). However, a lagged response of SSH, similar to the NAO, emerges if the variability in the winter mean wind stress curl and surface heat fluxes over the subpolar North Atlantic are considered (Fig. 6e,f). Although the pattern of the lagged SSH-response to surface heat fluxes resembles the lagged SSH-response to both the wind stress curl and the NAO, in this simulation, the role of winter mean surface heat flux variability is less important in driving decadal changes in SSH variability. This is because, in the model simulation, changes in surface density in the Labrador Sea are largely driven by changes in salinity and not by temperature (Fig. S2). Thus, the variability in surface heat fluxes over the subpolar North Atlantic would not lead to a lagged response in meridional overturning through changes in density. Therefore, these results suggest that the variability in SPG circulation as represented by SPGI (i.e. modelled PC1 SSH), in particular the latitudinal shift of the NAC in the western intergyre region, is a decadal response to wind stress curl variability over the subpolar North Atlantic.

## Discussion

The decadal meridional shifts in trajectories in the western intergyre region, which explain variability in modelled salinity, can originate from the variability in the NAO^24,25^. As a consequence of persistent multi-annual NAO+ state, the upper ocean heat content in the SPG is significantly lower and the SPG strengthens. As a compensating but delayed response, an anomalous anticyclonic intergyre gyre circulation is thought to build up in the western intergyre region as cooling in the SPG progresses^25^. However, in the present study, we demonstrate that the changes in the intergyre gyre are mainly driven by the variability in wind stress curl above the subpolar North Atlantic. Since the intergyre gyre circulation is anticyclonic for a strong SPG, the mean circulation would resemble a northward shifted NAC (Fig. 5a). While this is a local circulation anomaly outside of the core SPG, the build-up of this anomalous circulation is due to the same atmospheric forcing that drives the variability in the constituent currents of the core SPG circulation. The intergyre gyre circulation is thus conjectured to be induced by changes in SPG strength itself as a consequence of variability in wind stress curl.

Another mechanism that could explain decadal meridional shifts in the NAC is related to the variability in the AMOC. The dipole pattern of the modelled first EOF of SSH resembles the characteristic fingerprint of decadal AMOC variability^26^. The opposing pattern of SSH variability in the SPG and NAC path is a simple yet robust consequence of heat convergence/divergence in these regions in the MPI-ESM^27^. The flow of the deep western boundary current and its associated vortex stretching leads to the formation of a northern recirculation gyre in the upper layers in the NAC region^28^. This second mechanism also explains the anomalous circulation pattern in the western intergyre region and the associated weakening and strengthening of the SPG. The NAO is also indirectly involved in this mechanism since it influences both SPG temperature and the deep water formation in the Labrador Sea.

Although the meridional shift of trajectories in the intergyre region in Fig. 5b can be explained by the heat content related mechanisms discussed above, the broadening and shift of trajectories in the ENA can not be explained solely by either of the mechanisms. These mechanisms mainly emphasize external drivers of heat content change as the main source of variability in SPG circulation, however, they overlook the importance of salinity in influencing the stability of water column in the SPG (Fig. S2). This is because our results do not relate the variability in surface density in the Labrador Sea to changes in surface heat fluxes. Instead our results suggest that the initiation of the intergyre gyre circulation anomaly or the northern recirculation gyre in the western intergyre region, both of which relate to latitudinal shifts in the NAC, is due to wind stress curl driven changes in the SPG circulation.

When the vorticity input to the SPG due to winds is anomalously higher and persistent over multiple years, more fresh water is transported along the northern flanks of the NAC towards the ENA and less subpolar water flows southwards (Fig. 5a). Higher surface divergence associated with strengthened SPG circulation shifts major current pathways eastward, thus explaining the sharpening and eastward shift of simulated trajectories in the ENA. Thus a strong SPG contracts in the Newfoundland basin and expands in the ENA. A sustained removal of freshwater from the western SPG leads to a decrease in the thickness of fresh upper layer and consequently salinity increases over multiple years. Such salinification of western SPG creates positive density anomalies which propagate southwards and initiate positive anomalies in the meridional overturning circulation. The anomalous strengthening of the meridional overturning is concomitant with the westward contraction of the SPG in the ENA, the gradual retreat of the NAC towards southern latitudes and reversal of freshwater anomalies in the SPG.

The emergence of negative feedback mechanism in MPI-ESM-LR is consistent with earlier investigations in other models^29,30^, and points to a close coupling between horizontal and overturning circulation. However, our results suggest that the SPG is not passive in a perpetual negative feedback loop controlled by variability in the meridional overturning circulation. It is important to note that, as compared to the mean, the number of subtropical floats arriving in the ENA does not increase substantially when SPG weakens, but it decreases substantially when the SPG strengthens. The implication being that changes in the SPG size, strength and salinity in the ENA are not exclusively due to changes in subtropical throughput, rather, the variability in SPG circulation modulates the subtropical throughput which then changes the salinity in the ENA. These results are robust and not overly sensitive to the number and deployment depth of virtual floats (Figs. S3 and S4), Therefore, it is the variability in horizontal SPG circulation, driven by overlying atmospheric variability, that modulates the pathway and proportion of subpolar and subtropical floats arriving in the ENA, and such variability in the horizontal circulation is mainly captured by the modelled SPGI and to some extent DENS.

This result thus allows us to infer the likely cause of observed salinity changes in the ENA. Intensification of the NAC in the case of PC2 SSH, DENS, and NAO indices points to the increased advection of subpolar water from the west. The inverse relationship between observed subsurface density in the central SPG and salinity in the ENA supports this viewpoint, as does the response of circulation and salinity to long-term changes in the NAO (Fig. 3). Furthermore, in the Newfoundland Basin, the spatial pattern of geostrophic current difference between strong and weak circulation regimes matches with the modelled current composite difference. And since we showed through model experiments that the intergyre gyre circulation in the Newfoundland basin is a manifestation of expansion and contraction of the SPG, therefore, contrary to the recent finding^13^, the complex interplay between the variability in the strength and size of the SPG does influence salinity in the ENA. Our results also suggest that both PC2 SSH and DENS represent a dynamically consistent evolution of the SPG strength.

Having explored the causes of similarity between the SPGI-based and observed SPG-salinity relationship, we now highlight potential causes of discrepancies in modelled FKL and BTSF. In the model, the SPG does expand and contract, however, if one considers the largest closed contour of SSH as the SPG boundary, then the zonal expansion and contraction of the SPG is limited to the western parts (Fig. S5). By definition, this would exclude variability in the ENA and in the western intergyre region, which as discussed above is coupled to the SPG circulation via atmospheric or oceanic forcing, and therefore FKL neither projects onto NAO nor shows consistent relationship with salinity in the ENA^13^. From the model point of view, the SPG is a cyclonic circulation seen in the two dimensional barotropic stream function in the subpolar North Atlantic. The area and depth averaged BTSF as one of the indices analyzed here only partly captures the complexity of the three dimensional spatial structure of SPG circulation. Furthermore, the BTSF represents circulation strength in the western SPG (Fig. S5), and does not capture the baroclinic component of circulation variability in the intergyre region and the ENA either.

While the results presented here illuminate the SPG-salinity relationship in observations and in an ESM, there are some caveats that must be acknowledged. First, as is the case with various global coupled models, there are known issues in MPI-ESM regarding the zonal position of NAC^31^. This leads to biases in mean water mass properties. Advection of such biased properties to regions of interest is a concern. Hence, the potential impact model biases could have on the variability of SPG circulation warrants further investigation. Second, the altimeter record is quite short (24 years) compared to the model output (200 years) analyzed here. Thus, the low frequency variability present in modelled SPG indices might not have yet emerged in the observed indices, and thus their comparison must be treated with caution. Finally, given the scarcity of long term velocity observations, we have only analyzed the baroclinic part of observed circulation variability. Since the barotropic part of circulation variability is not in phase with the baroclinic part^14^, the changes in observed circulation presented in this study must also be carefully interpreted. Nevertheless, the similarity between the response of modelled currents and the observed baroclinic currents suggests that the baroclinic part of circulation variability plays an important role in determining SPG strength-salinity relationship in the ENA.

## Conclusions

Based on the analysis of observed and modelled SPG indices and Lagrangian trajectory experiments carried out with a global coupled model, we derive the following conclusions:

1. The interpretation of the SPG strength–salinity relationship is dictated by the choice of the SPG index. A dynamically consistent interpretation presented here is that the variability in the proportion and the pathway of subpolar water arriving in the ENA has an influence on salinity. Thus both size and strength of the SPG are related to salinity changes in the ENA.
2. All modelled SPG Indices agree that a stronger SPG is associated with enhanced influence from the fresher western SPG region and impedes the subtropical salinity throughput.
3. The modelled variability in Lagrangian pathways is part of the decadal response of SPG circulation to wind stress curl variability over the subpolar North Atlantic. Latitudinal shifts of the NAC in the western intergyre region and pathways of subpolar water in the ENA are most likely main sources of inconsistency among SPG indices.
4. Constrained by their definitions, SPG indices based on depth integrated barotropic streamfunction and the largest closed contours of SSH should be carefully interpreted as these indices do not capture the variability in the advective pathways from the western intergyre region to the ENA.
5. SPG indices based on principal component analysis of SSH and subsurface density capture the variability in the strength and position of the NAC and the intensification of cyclonic circulation in Irminger Sea and Iceland basin. These indices are therefore the best-suited proxies of SPG circulation variability and associated water mass variability in the ENA.

Finally, the weak-SPG-high-salinity relationship in the eastern subpolar North Atlantic is a robust basis for the choice of SPG indices in observations and models.

## Methods

Observations of annual mean salinity used in this study are taken from the EN4^32^ gridded dataset and cover the time period 1960–2016. Potential density (*σ*_0_) is calculated from temperature (T) and salinity (S) from the EN4 dataset. We also calculate geostrophic velocities to examine variability in the baroclinic part of observed circulation variability. The geostrophic velocities were calculated from the T and S fields of the EN4 dataset using the geostrophic equation and dynamic topography, while assuming a layer of no motion at 1500 m depth (results do not change substantially if this depth is changed to other reasonable level). The horizontal gradients and velocity components were calculated at the mid point of the original grid boxes. The monthly mean SSH data (i.e., AVISO absolute dynamic topography on a 0.25° × 0.25° grid) was obtained from the Integrated Climate Data Center (http://icdc.cen.uni-hamburg.de/1/daten/ocean/ssh-aviso.html), and covers the time period 1993–2016. Annual means were calculated from monthly mean values.

The model used in this study is the Max Planck Institute Earth System Model, run in a low resolution configuration (MPI-ESM-LR^33^). The ocean component of MPI-ESM-LR has a nominal resolution of 1.5 degrees with a gradual increase in resolution towards the subpolar North Atlantic, reaching approximately 18 km near Greenland. We analyzed the last 200 years of a 2000-year pre-industrial control simulation where the model is free to evolve dynamically. This type of simulation is neither initialized from observation nor forced with observed external boundary conditions. This enabled us to examine SPG-salinity relationship in a dynamically consistent setting. The model output was regridded to a 1° × 1° regular grid and annual mean values were analyzed in composite analysis. The model output does not exhibit any significant trend, so we present results based on the non-detrended model output. Detrending does not result in any different conclusions.

In the present work, we used multiple indices of SPG strength that have been applied in observational and modelling studies (Table S1). The first index (PC1 SSH) is defined as the principal component of the leading Empirical Orthogonal Function (EOF) of annual mean SSH anomalies in the subpolar North Atlantic, defined in the domain 20°N to 70°N, 0°W to 80°W^5^, and defined for the altimeter period 1993–2016. Similarly, the second index (PC2 SSH) is defined as the principal component of the second EOF of annual mean SSH anomalies. A third index (DENS) is defined as the annual mean density anomalies at 310 meter depth in the region 50°N to 62°N, 35°W to 55°W^20^. DENS is a proxy for baroclinic strength of the SPG previously used to explain freshening in the western SPG. DENS is defined for two periods: the altimeter period 1993–2016, for comparison with other SSH-based indices, and 1960–2016, to extend the analysis for a longer time period. A fourth index (FKL) is defined as the difference between the largest closed contour of annual mean SSH and the minimum of SSH within the largest closed contour^13^. The FKL index is defined for the altimeter period from 1993 to 2016. Additionally, we also use two pentads when the NAO was largely in a high (1991–1995) or a low (1964–1968) state as a proxy of those atmospheric conditions which favor a particular SPG state. The observed NAO index is the Hurrel’s station-based index and is obtained from https://climatedataguide.ucar.edu/climate-data/. All observational results presented in this study are based on non-detrended data.

The SPG indices - PC1 SSH, DENS and FKL - defined using observational data are defined identically in the MPI-ESM. Another index (BTSF) is defined in MPI-ESM as the annual mean depth integrated barotropic streamfunction in the region 50°N to 62°N, 10°W to 60°W. Our region of interest for identifying salinity changes is the eastern subpolar North Atlantic (ENA), defined as the region between 50°N to 60°N, 15°W to 30°W.

In order to test whether the variability in advective pathways leads to variability in salinity in the ENA, we performed Lagrangian experiments in MPI-ESM using the offline OceanParcels^34,35^ framework for the particle trajectory scheme. First, composite mean monthly horizontal and vertical velocity fields based on strong and weak phases of each index were created. Then the resulting monthly mean 3-dimensional velocity field was used to advect 500 virtual floats deployed randomly in the upper 100 meters in the ENA (50°N to 60°N, 15°W to 30°W) and advected backward in time for four years. The interested reader is referred to http://oceanparcels.org/ for more details on the underlying particle trajectory scheme.

## Supplementary information

Supplementary Info.

## Data Availability

The Observational data sets used in this study are publicly available. The model data analyzed in this study is available from the corresponding author upon reasonable request or are accessible at the DKRZ (www.dkrz.de/up).
