# Supplementary material for: Unraveling the choice of the north Atlantic subpolar gyre index
Source: Sci Rep. 2020 Jan 22;10:1005. doi: 10.1038/s41598-020-57790-5 (PMC6976698; doi:10.1038/s41598-020-57790-5)
Supplement: Supplementary file 1 — Supplementary Info. [file 41598_2020_57790_MOESM1_ESM.pdf]

Supplementary Information for "Unraveling the choice of the north Atlantic subpolar gyre index"

Vimal Koul[1,2,3], Jan-Erik Tesdal[4], Manfred Bersch[1], Hjálmar Hátún[5], Sebastian Brune[1], Leonard Borchert[1,6,7], Helmuth Haak[6], Corinna Schrum[3]and Johanna Baehr[1]

[1]Institute of Oceanography, Center for Earth System Sustainability, Universität Hamburg, Germany

[2]International Max Planck Research School on Earth System Modelling, Max Planck Institute for Meteorology, Germany

[3]Helmholtz Zentrum Geesthacht, Institute of Coastal Research, Germany

[4]Lamont-Doherty Earth Observatory, Columbia University, Palisades, New York

[5]Faroe Marine Research Institute, Faroe Islands

[6]Max Planck Institute for Meteorology, Germany

[7]Sorbonne Universités (SU/CNRS/IRD/MNHN), LOCEAN Laboratory, Institut Pierre Simon Laplace (IPSL), Paris, France

Contents of this file

1. Text S1
2. Figures S1 to S5
3. Tables S1 to S2

S1 Composite Analysis

Due to different lengths of observed and modelled time series, for the composite analysis, we have chosen values above and below ± 0.5 standard deviation for observation-based SPG indices and ± 1 standard deviation for model-based SPG indices. This choice of these separate thresholds is guided by the necessity to minimize any bias in the number of strong and weak SPG years. We find that by using the thresholds as they are in this study, this requirement is best fulfilled. We have also compared the observational results based on ± 1 standard deviation, and we find that the conclusions derived in this study do not change. Statistical significance tests performed in the supplementary figures presented below follow the methodology of Koul, Schrum, Düsterhus, and Baehr (2019).

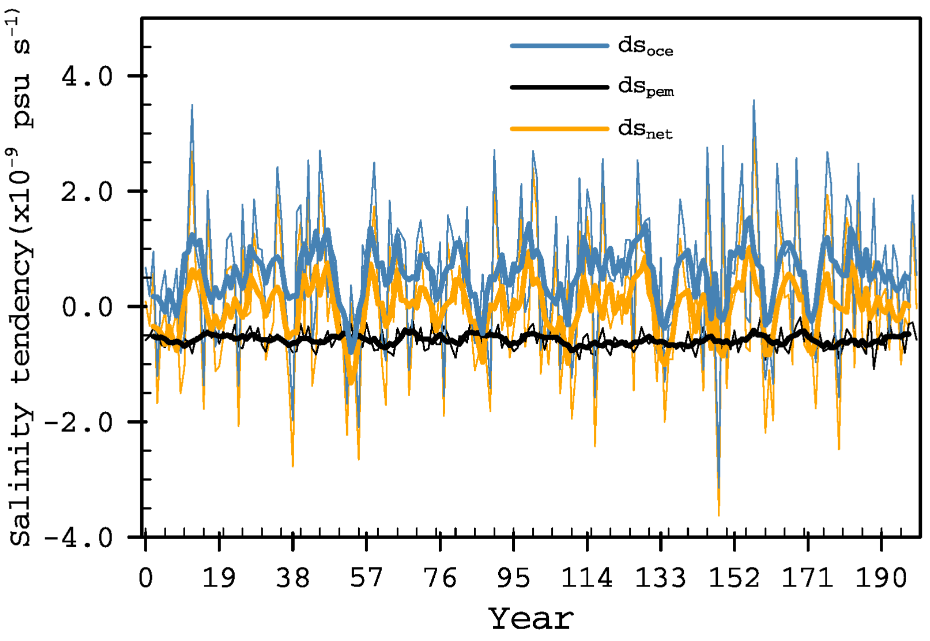

Figure S1. Contribution to net salinity tendency (ds_{net}, orange) in the upper 500m in the ENA from precipitation minus evaporation (ds_{pem}, black) and ocean (ds_{oce}, blue). This salt budget is carried out for the last 200 years of the model simulation. The contribution from the ocean includes advection, diffusion and the salt flux from the bottom of the control volume.

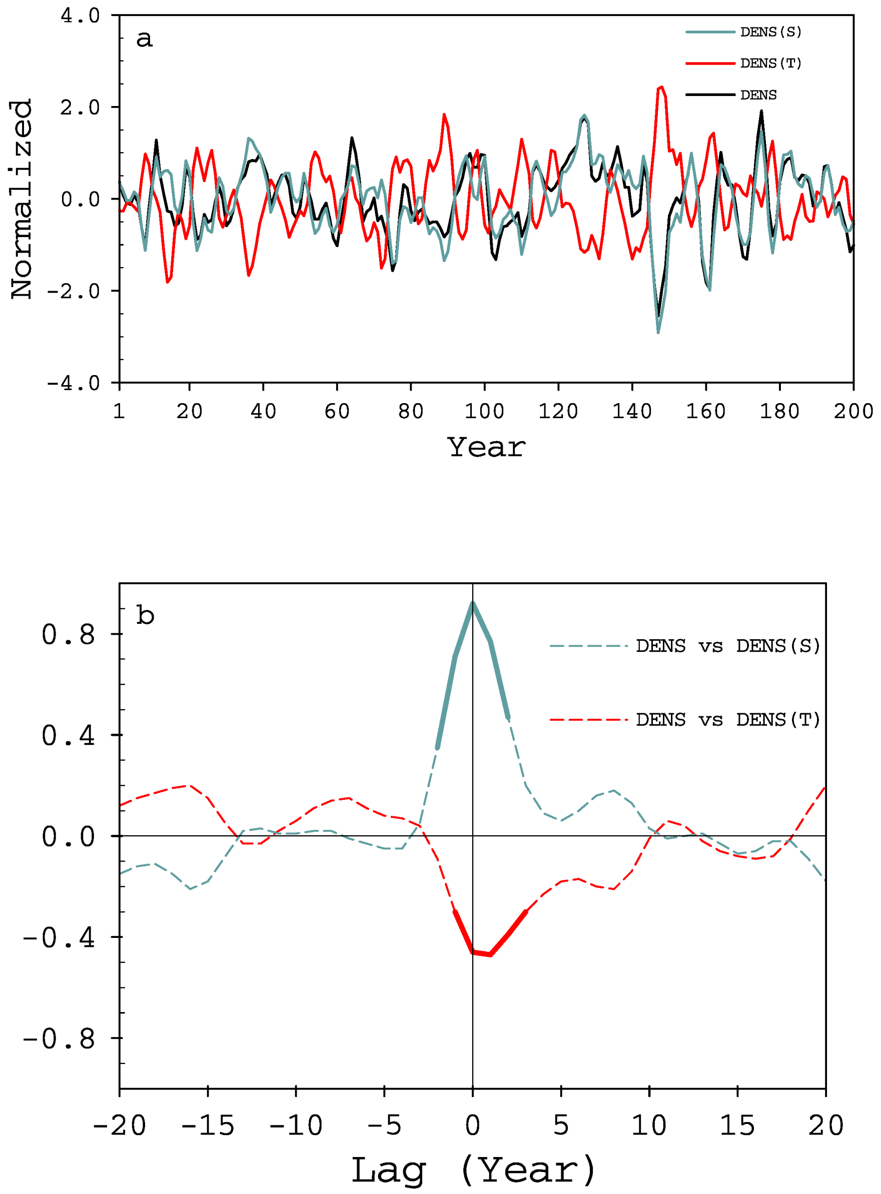

Figure S2. (a) Time series of modelled surface density (black), density due to variable salinity and mean temperature (blue) and density due to variable temperature and mean salinity (red) in the Labrador Sea (55:65N, 45:60W) for the last 200 years of the simulation. (b) Lead-lag correlation between density and density due to variable salinity (blue) and density due to variable temperature (red). Density leads for positive lags. Statistically significant correlations at 95% confidence level are shown in bold. All three time series were smoother by a 3-year running mean before correlating.

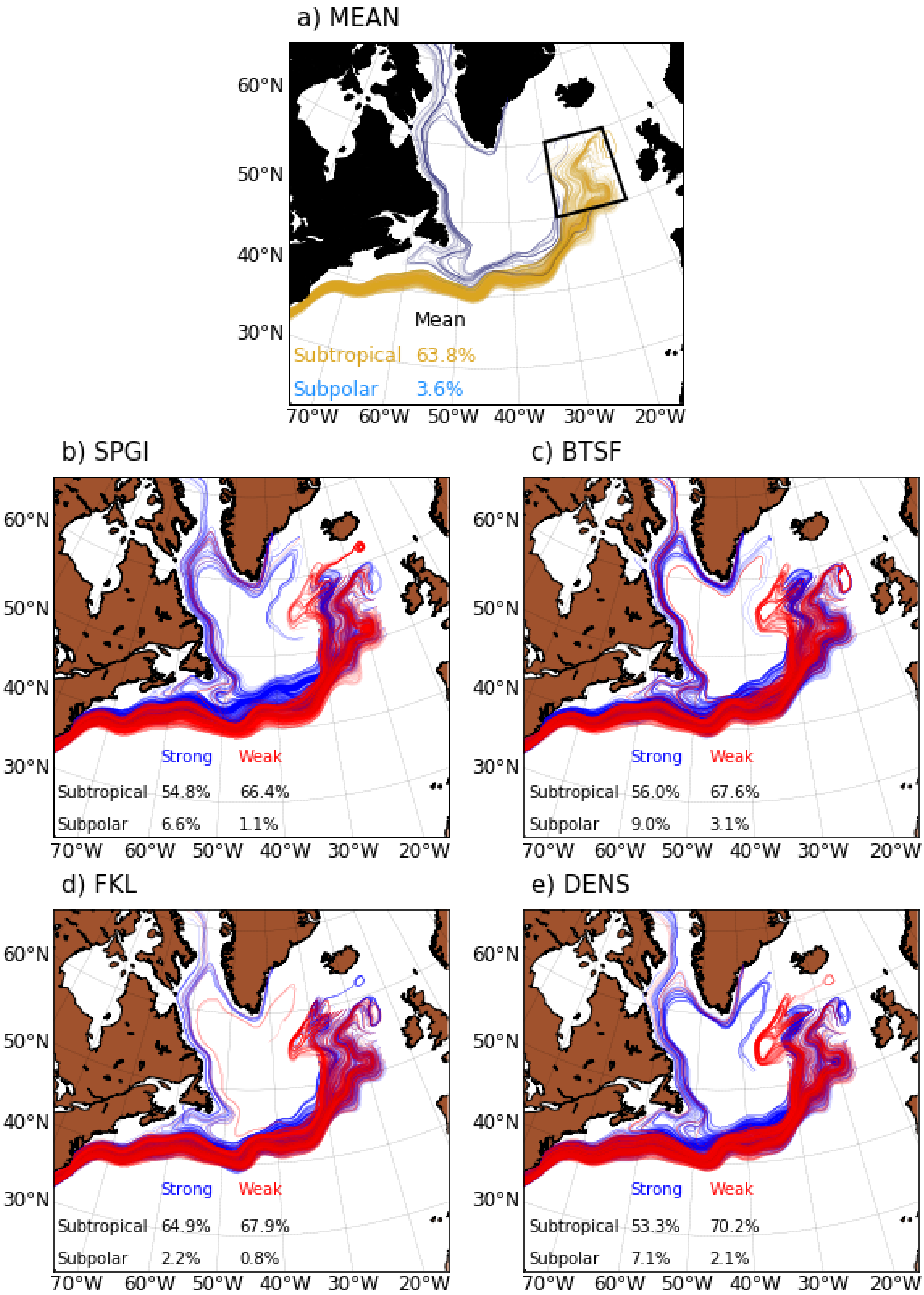

Figure S3. Same as Figure 5 in the main text but for 1000 floats deployed in the upper 100m.

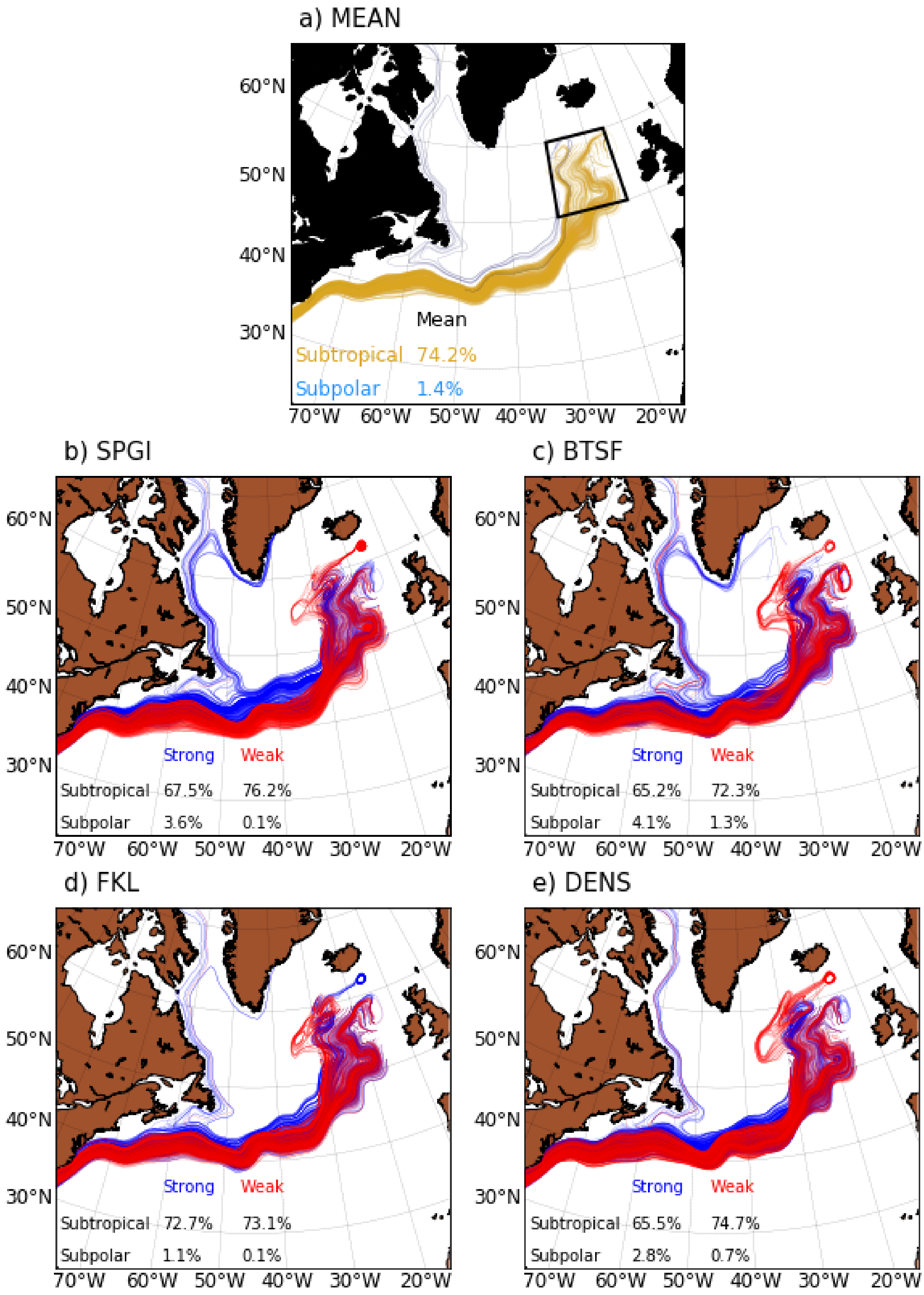

Figure S4. Same as Figure 5 in the main text but for 1000 floats deployed in the upper 200m.

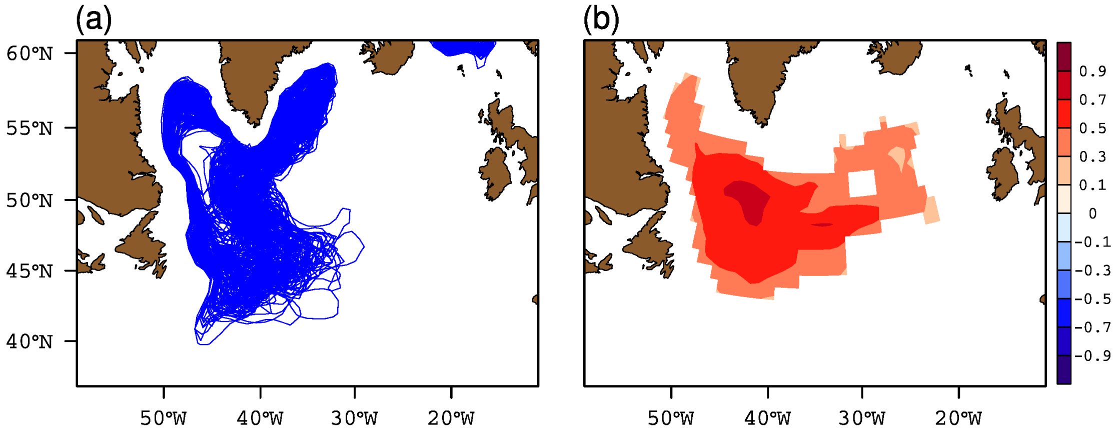

Figure S5. (a) Largest closed contour of modelled annual mean SSH for each year of the simulation period. (b) Correlation of BTSF index with the annual mean barotropic streamfunction at each grid point. Statistically significant values at 95% confidence level using a t-test are shown.

Table S1. Indices of SPG strength

	DEFINITION	TIME PERIOD	REFERENCE
PC1-SSH	Principal Component of the first empirical orthogonal function of sea surface height in the North Atlantic (20:70°N, 0:80°W	Observation: 1993:2016<br>Model: Last 200 years	Häkkinen and Rhines (2004); Hátún, Sandø, Drange, Hansen, and Valdimarsson (2005)
PC2-SSH	Principal Component of the second empirical orthogonal function of sea surface height in the North Atlantic (20:70°N, 0:80°W	Observation: 1993:2016<br>Model: Last 200 years	As also used in Hátún and Chafik (2018)
DENS	Weighted area average density anomaly at 314 m depth in the region:50:62°N, 35:55°W	Observation: 1993:2016<br>Model: Last 200 years	Tesdal, Abernathey, Goes, Gordon, and Haine (2018)
FKL	Difference between the largest closed contour of annual mean SSH in the subpolar North Atlantic and minimum of SSH within the largest closed contour.	Observation: 1993:2016<br>Model: Last 200 years	Foukal and Lozier (2017)
BTSF	Weighted area average barotrophic streamfunction in the North Atlantic (50:62°N, 10:60°W)	Model: Last 200 years	As also used in Lohmann, Drange, and Bentsen (2009)

Table S2. Strong and weak years (from non-detrended data) of SPG strength

PC1 SSH		PC2 SSH		DENS		FKL	
Strong	Weak	Strong	Weak	Strong	Weak	Strong	Weak
1993	2012	1993	2003	1993	1996	1993	2004
1994	2013	1994	2004	1994	1998	1995	2005
1995	2014	1995	2005	1995	1999	1996	2009
1996	2015	2000	2006	2000	2002	1997	2010
1997	2016	2015	2007	2015	2003	1998	2016
		2016	2010	2016	2004	2002	
			2011		2006	2007	
			2013		2007	2014	
					2011		

References

Foukal, N. P., & Lozier, M. S. (2017). Assessing variability in the size and strength of the N orth A tlantic subpolar gyre. *Journal of Geophysical Research: Oceans*, *122*(8), 6295–6308.

Häkkinen, S., & Rhines, P. B. (2004). Decline of subpolar North Atlantic circulation during the 1990s. *Science*, *304*(5670), 555–559.

Hátún, H., & Chafik, L. (2018). On the recent ambiguity of the North Atlantic subpolar gyre index. *Journal of Geophysical Research: Oceans*.

Hátún, H., Sandø, A. B., Drange, H., Hansen, B., & Valdimarsson, H. (2005). Influence of the Atlantic subpolar gyre on the thermohaline circulation. *Science*, *309*(5742), 1841–1844.

Koul, V., Schrum, C., Düsterhus, A., & Baehr, J. (2019). Atlantic inflow to the north sea modulated by the subpolar gyre in a historical simulation with mpi-esm. *Journal of Geophysical Research: Oceans*, *124*(3), 1807–1826.

Lohmann, K., Drange, H., & Bentsen, M. (2009). A possible mechanism for the strong weakening of the North Atlantic subpolar gyre in the mid-1990s. *Geophysical Research Letters*, *36*(15).

Tesdal, J.-E., Abernathey, R. P., Goes, J. I., Gordon, A. L., & Haine, T. W. (2018). Salinity trends within the upper layers of the subpolar North Atlantic. *Journal of Climate*, *31*(7), 2675–2698.
